# Bioinformatic Analysis of the BTB Gene Family in *Paulownia fortunei* and Functional Characterization in Response to Abiotic and Biotic Stresses

**DOI:** 10.3390/plants12244144

**Published:** 2023-12-12

**Authors:** Peipei Zhu, Yujie Fan, Pingluo Xu, Guoqiang Fan

**Affiliations:** 1 College of Forestry, Henan Agricultural University, Zhengzhou 450002, China; 13730357685@163.com (P.Z.); fanyujie@henau.edu.cn (Y.F.);; 2 Institute of Paulownia, Henan Agricultural University, Zhengzhou 450002, China

**Keywords:** *Paulownia fortunei*, BTB, miRNA, gene expression, Paulownia witches’ broom, environmental responses

## Abstract

To learn about the gene structure, phylogenetic evolution, and function under biotic and abiotic stresses of BTB (Bric-a-Brac/Tramtrack/Broad Complex) genes in *Paulownia fortunei*, a whole-genome sequence evaluation was carried out, and a total of 62 *PfBTB* genes were identified. The phylogenetic analysis showed that PfBTB proteins are divided into eight groups, and these proteins are highly conserved. *PfBTB* genes were unevenly distributed on 17 chromosomes. The colinearity analysis found that fragment replication and tandem replication are the main modes of gene amplification in the *PfBTB* family. The analysis of *cis*-acting elements suggests that *PfBTB* genes may be involved in a variety of biological processes. The transcriptomic analysis results showed that *PfBTB3/12/14/16/19/36/44* responded to Paulownia witches’ broom (PaWB), while *PfBTB1/4/17/43* responded to drought stress, and the RT-qPCR results further support the reliability of transcriptome data. In addition, the association analysis between miRNA and transcriptome revealed a 91-pair targeting relationship between miRNAs and *PfBTBs*. In conclusion, the *BTB* genes in *Paulownia* are systematically identified in this research. This work provides useful knowledge to more fully appreciate the potential functions of these genes and their possible roles in the occurrence of PaWB and in response to stress.

## 1. Introduction

Currently, the BTB protein has been identified in many plants, including *Arabidopsis thaliana* [1,2,3], rice [4], tomato [5], corn [6], and peach [7]. In *Arabidopsis thaliana*, BTB proteins can be divided into different subfamilies based on their contained conserved domains other than the BTB domain, including BTB-MATH, BTB-Arm, BTB-only, BTB-TAZ, BTB-BACK, BTB-Ank, TPR, BTB-NPH3, and other [8,9,10,11,12,13]. The BTB (Bric-a-Brac/Tramtrack/Broad Complex) domain, also known as POZ (pox virus and zinc finger), is a conserved sequence of about 115 amino acids [14]. This domain was first discovered in the Bric-a-Brac, Tramtrack, and Broad Complex proteins of fruit flies (Drosophila melanogaster) [15]. 

In plants, proteins containing the BTB domain can participate in transcriptional inhibition, protein ubiquitination degradation, and signal transduction of plant hormones and play a critical role in stress and disease resistance [16,17]. In apples, the BTB family protein, *MdBT2*, promotes the ubiquitination and degradation of *MdARF8* through the 26S proteasome pathway, negatively regulating the expression of *MdGH3.1* and *MdGH3.6*, thereby inhibiting the formation of lateral roots [18]. Previous studies have shown that ETO1, EOL1, and EOL2 in the BTB family of *Arabidopsis thaliana* can regulate ethylene synthesis [19]. Systemic acquired resistance (SAR) in plants is mediated by the *Arabidopsis thaliana* BTB gene NPR1, which functions as a salicylic acid (SA) receptor [20,21]; when it is overexpressed in plants, resistance to biotic and abiotic stresses will be enhanced. As a homolog of NPR1, *OsNPR1/NH1* contributes to rice’s resistance to *Xanthomonas oryzae pv-caused* bacterial blight [22]. In transgenic tomatoes, *AtNPR1* overexpression exhibits broad-spectrum resistance to pathogens [20]. Research has shown that the SA receptors, NPR1 and NPR3/NPR4, in *Arabidopsis thaliana* have opposing functions in the transcriptional regulation of plant immunity [23]. Furthermore, the expressions of *AtNPR1* in tomatoes and wheat enhances the resilience of the tomato plant to bacterial and fungal infections and the defense response against *Fusarium head blight* (*FHB*), respectively [20,24]. Similarly, *GmBTB/POZ* in soybean also plays a role in the plant response to Phytophthora soybean; its overexpression in this plant enhances its resistance to pathogen infection [25]. In addition, cold, drought, and salt stress control the expression of the cucumber BTB gene, which displays various patterns of expression in cucumber tissues [26]. These findings suggest that plant BTB genes regulate biotic and abiotic stress responses.

Paulownia is an important deciduous tree because of its fast growth and excellent wood, and it often is used in construction and making furniture and musical instruments and helps alleviate the shortage of wood. Its flowers, leaves, fruits, and bark can be used as medicine, as it has a high medicinal value. Paulownia has strong adaptability and resistance to improve the soil and ecological environment and manage saline and alkaline land [27]. Nevertheless, paulownias are susceptible to phytoplasma infection, leading to the occurrence of Paulownia witches’ broom (PaWB). Due to susceptibility to phytoplasma infection, the resulting symptoms include axillary bud clusters, plant dwarfing, and yellow flowers on leaves, causing slow growth or even death of large trees, which seriously affects the yield and economic benefits of paulownias [28]. This infection causes the plant to have overgrown axillary buds and dwarfism, which increase tree mortality, slow tree growth, and seriously affect the growth of *Paulownia* production. Therefore, it is important to identify the relevant genes against the occurrence of PaWB in *Paulownia*. At present, studies on the molecular mechanism of the pathogenesis of PaWB have made great progress [27,29,30,31]. The genomes of both *Paulownia fortunei* and PaWB phytoplasma have been decoded [27]. Despite the former, the members of the *PfBTB* gene family in this species have not yet been reported. Since the BTB family plays a crucial role in biological stress responses, it is very important to identify it. Based on the *P. fortunei* genome sequence, we can better understand the possible functions of BTB genes in PaWB. In this study, we adopt a bioinformatics approach to identify members of the BTB gene family on a genome-wide scale, investigating the physico-chemical properties, phylogenetic relationships, genetic structure, and *cis*-regulatory element and their expressions when stressed by biotic and abiotic factors and hormonal treatments. Altogether, our research provides the candidate BTB genes involved in PaWB response and stress-related pathways, providing a foundation for the genetic breeding of paulownias. At the same time, it provides new ideas and directions for the prevention and control of PaWB.

## 2. Results

### 2.1. Understanding the PfBTB Gene Family and Examining the Properties of Proteins

There were 62 genes found in the *P. fortunei* genome that were possible BTB gene family members. Then, we named these identified *PfBTB* genes according to the chromosomal location (Table 1). The *PfBTB* gene family has a molecular weight (MW) of 24.88 to 115.07 kDa (Kilodalton) and an isoelectric point (pI) of 4.64 to 9.52. Cell-PLoc 2.0 predicted the subcellular location of *PfBTB*s, and it was determined that the majority of *PfBTBs* were located in the nucleus (Table 1).

### 2.2. Analysis of the Phylogenetic Tree of Members of the PfBTB Gene Family

**Figure 1:**
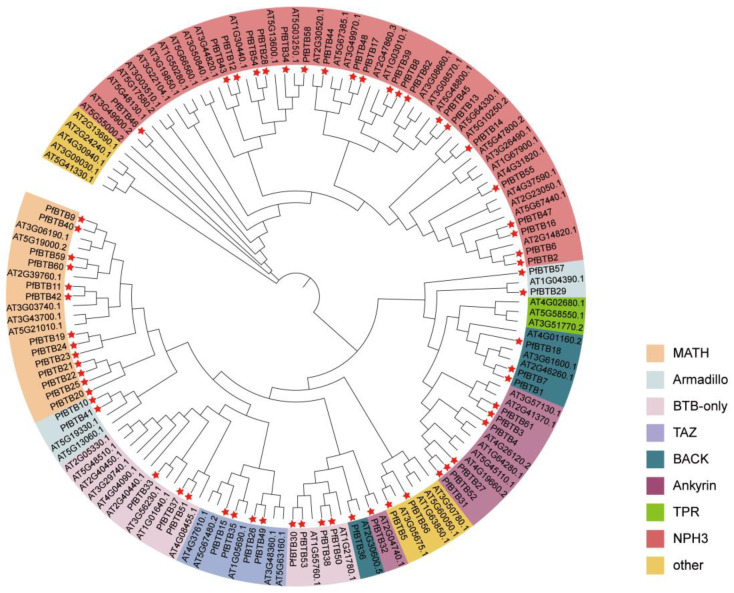
The phylogenetic tree of the *PfBTB* gene family. A phylogenetic tree of the BTB proteins of the two species, *P. fortunei* and *Arabidopsis thaliana*. MEGA-X 10.2 software was used to build the phylogenetic tree using 1000 bootstraps. The red pentagon star is used to represent the BTB protein in *P. fortunei*. Two species contain all 142 BTB proteins that were clustered into 9 subgroups, named MATH, Armadillo, BTB-only, TAZ, BACK, Ankyrin, TPR, NPH3, and other.

To examine the evolutionary relations of the *PfBTB* gene family, we manufactured a phylogenetic tree (NJ, neighbor-joining) using the MEGA-X 10.2 tool based on the BTB proteins with *P. fortunei* (62 members) and *Arabidopsis thaliana* (80 members) (Figure 1). The phylogenetic tree’s findings indicate that 142 members of the BTB genes from the 2 species were gathered into 9 sub-groups, containing Ankyrin, MATH, Armadillo, BTB-only, BACK, TAZ, TPR, NPH3, and other. The number of BTB proteins between *P. fortunei* and *Arabidopsis thaliana* varied greatly in the same subfamily. The NPH3 subfamily has the largest number, including 21 *Paulownia* BTB proteins and 32 *Arabidopsis thaliana* BTB proteins (Figure 1).

### 2.3. Gene Structure and Conserved Motif Analysis

**Figure 2:**
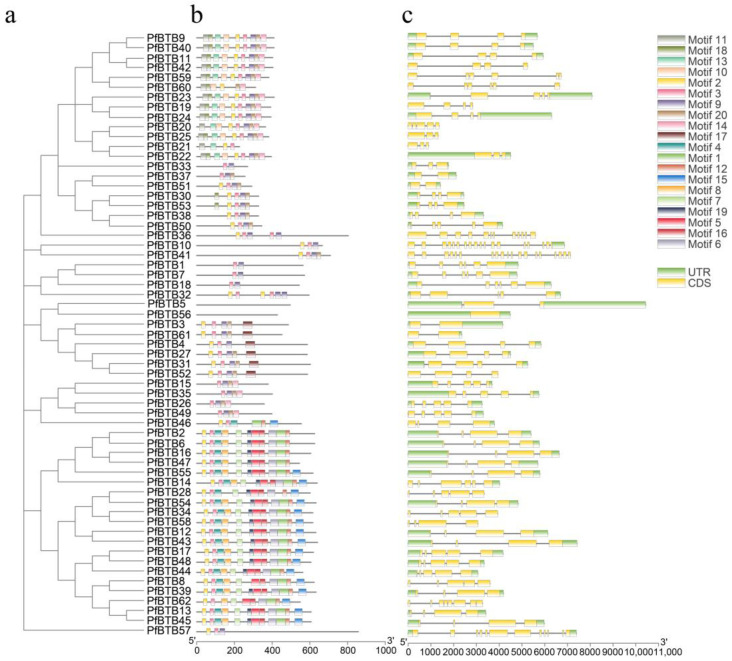
The investigation of the conserved motifs of the *P. fortunei* BTB gene family’s genes. (**a**) Phylogenetic tree constructed using 62 *PfBTB* genes and neighbor-joining (NJ); (**b**) Examination of *P. fortunei*’s BTB genes’ conserved motifs. The MEME tool generated a total of 20 motifs, designated Motif 1–20. 200 aa is indicated by the scale bar; (**c**) Analysis of the UTR, intron, and exon regions in *P. fortunei*’s BTB genes. UTR is represented by the green rectangles, Exon by the yellow rectangles, and Intron by the grey lines. The scale bar shows a value of 2 kb.

To further substantiate the evolutionary tree’s classification findings, we looked at the conserved motifs and gene architectures of *P. fortunei*’s *PfBTB* genes. The MEME suite (http://meme-suite.org/tools/meme (accessed on 13 September 2022)) was utilized to estimate 20 motifs, and we designated motifs 1 through 20 (Figure 2). Among them, Motif 2 was found among all clusters, signifying that it is highly conserved in all PfBTB proteins. According to the predicition analysis on the SMART website, it was found that the BTB domain is composed of Motif 1, 3 and 5. This result provides evidence of our accuracy in studying the BTB gene.

To investigate the *PfBTB* gene structures of *P. fortunei*, from the *P. fortunei* database, we retrieved the exon–intron information for 62 *PfBTB* genes. Based on this information, the TBtools software (v. 2.019) was used to display these structures (Figure 2). The exon counts in *PfBTB* genes varied widely, ranging from 1 to 19. *PfBTB41* has the most exons (19) out of the 62 BTB genes in Paulownias, while 26 BTB genes have four exons (41.93%).

Additionally, the exon and intron lengths were different. A total of 49 BTB genes were discovered to contain untranslated regions (UTR). Similar gene architectures among the BTB genes were clustered into one subclade, mirroring the results of the motif analysis. This finding demonstrates the possibility of the same groups sharing genomic architecture and conserved motifs across group members. These findings offer compelling proof that the results of the phylogenetic tree classification are accurate.

### 2.4. PfBTB Gene Locations on the Chromosome and Homologous Gene Analysis

**Figure 3:**
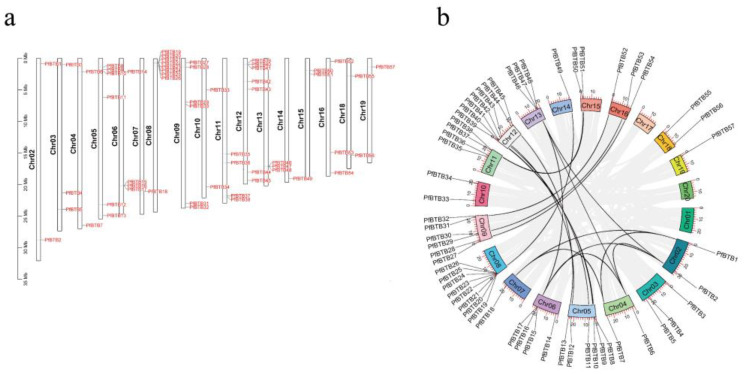
The distribution of BTB genes in *P. fortunei*’s genome based on location and synteny analysis. (**a**) The location of *PfBTB*s on each of the 20 *P. fortunei* chromosomes. Chromosomes 1 and 20 do not contain any BTB genes, hence we did not display them in Figure 3.; (**b**) The BTB gene family’s distribution pattern synteny study. The synteny gene pairs of the BTB gene family are shown by the black lines.

TBtools (v. 2.019) was used to anticipate the *PfBTB* genome’s chromosomal distribution pattern (Figure 3a). Paulownias were used to derive the BTB gene’s location information. The results showed that no members of this gene family were mapped to chromosomes 1, 17, or 20, and that 57 *PfBTB* genes (91.93%) were unevenly distributed among the 20 Paulownias chromosomes. Therefore, we did not show them in our study. In addition, five other genes that were situated on scaffold contigs were likewise not displayed. Eight BTB genes were found on chromosome 8, with seven genes on chromosome 12 coming in second. Six BTB genes were located on chromosomes 5 and 9. There were four BTB genes on each of chromosomes 6 and 11. On chromosomes 2, 3, 4, 10, 13, 15, 16, and 18, two or three BTB genes were located. Chromosomes 7, 14, and 19 contained a single gene. Using the MCScanX program, we also located the homologous genes of the BTB gene family. The results show that the BTB gene family of paulownias, which has 37 identical genes, contained 24 homologous gene pairs. Between chromosomes 9 and 16, there were three homologous gene pairs (Figure 3b).

### 2.5. Cis-Acting Element Prediction of PfBTB Genes

**Figure 4:**
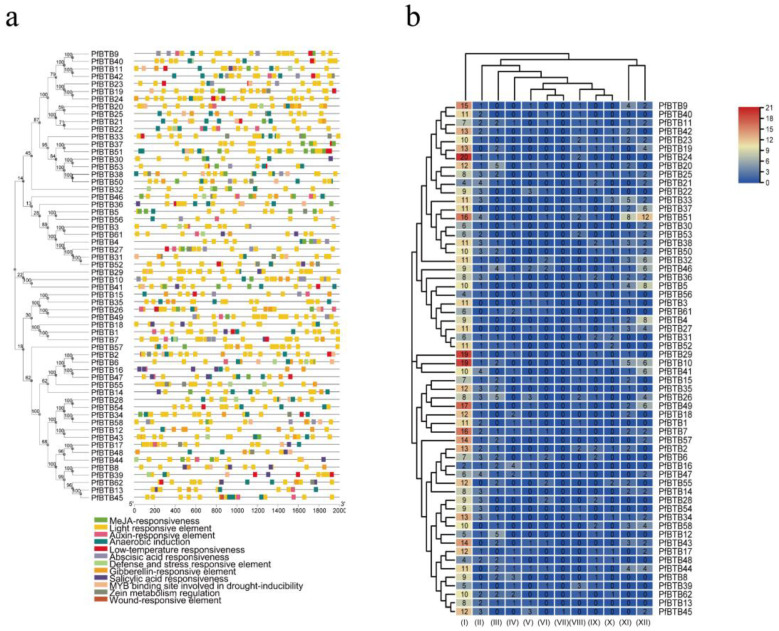
Analysis of the potential promoter of *PfBTB* genes’ *cis*-acting elements. (**a**) There are 12 *cis*-acting elements in the putative *PfBTB* gene promoter. The color scale at the top right indicates the number of *cis*-acting components. The color represents the wide range of *cis*-acting influences on BTB members. There are 12 *cis*-acting components, including the following: (I) light responsive element; (II) anaerobic induction; (III) gibberellin-responsive element; (IV) salicylic acid responsiveness; (V) auxin-responsive element; (VI) defense and stress responsive element; (VII) wound-responsive element; (VIII) low-temperature responsiveness; (IX) MYB binding site involved in drought-inducibility; (X) zein metabolism regulation; (XI) abscisic acid responsiveness; (XII) MeJA-responsiveness. (**b**) The distribution pattern of 12 *cis*-acting regions of the BTB gene family’s putative promoter in *Paulownia*.

*Cis*-acting elements are necessary clues in predicting gene functions. By attaching to the *cis*-acting component of target genes in particular organic processes, transcription elements may affect the level of gene expression [32]. In addition, to further study the function of the *PfBTBs*, we used the Plant CARE database to predict the *cis*-acting elements of the promoter regions. As a consequence, 62 *cis*-acting elements were recognized, of which we chose 12 interesting ones for more research. These are related to stress, hormones, and plant growth and development. According to Figure 4a, numerous distribution patterns of *cis*-acting elements were discovered in the promoter region of *PfBTB* genes, demonstrating the importance of the BTB gene family of *Paulownia* in a range of biological processes. In the meanwhile, we discovered that all *PfBTB* genes contain the cis-acting elements associated with the regulation of hormones, including responsiveness factors to salicylic acid (SA), gibberellin (GA), auxin (IAA), and methyl jasmonate (MeJA). The ABA-responsive element, or ABRE, is one of the most crucial cis-acting elements in the promoter sequence that controls the expression of the ABA-inducible gene in response to ABA therapy [33]. In total, 37 *PfBTB* genes were identified in our analysis as ABA responsiveness factors, indicating that the BTB gene family may be involved in ABA signal transduction (Figure 4b). Additionally, 30 *PfBTB* genes were found to contain cold-related *cis*-acting regions, suggesting that they might have particular resistance under low-temperature treatment.

### 2.6. PfBTB Genes Response to Phytoplasma and Expression Patterns in Different Tissues/Organs

PaWB infection is one of the main causes of *P. fortunei* mortality [27]. Studies have revealed that the BTB gene has a significant defensive role against biological stress. To identify key disease resistance genes, we examined the amounts of BTB gene transcription in PaWB-infected *P. fortunei* (PFI) and healthy *P. fortunei* (PF). From the transcriptome determined in the laboratory, we found that 7 of the 62 *PfBTB* genes are significantly different in PF/PFI, where *PfBTB12*, *PfBTB14*, and *PfBTB44* were significantly upregulated and *PfBTB3*, *PfBTB16*, *PfBTB19*, and *PfBTB36* were significantly downregulated.

**Figure 5:**
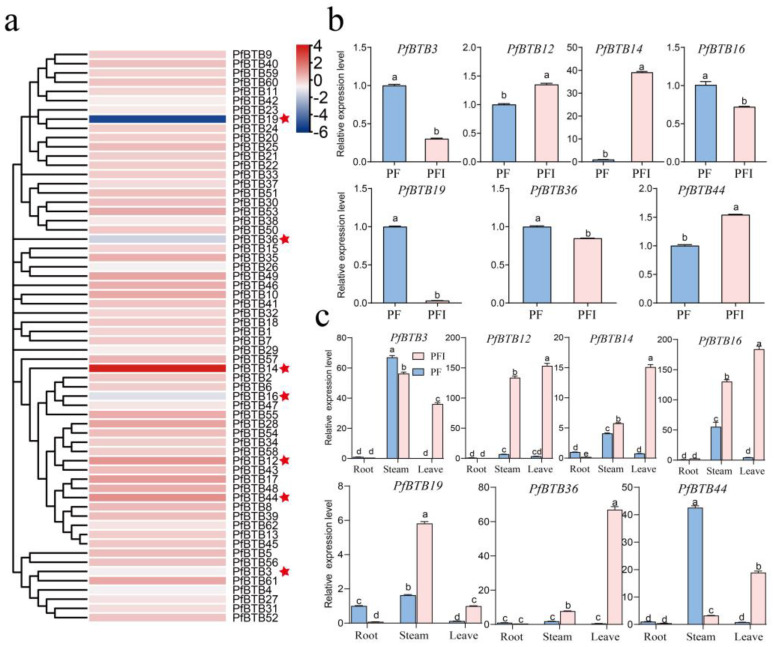
Expression levels of BTB gene family members of *Paulownia foutunei* in healthy and phytoplasma-infected seedlings. (**a**) Heat maps are plotted using log_2_RPKM and 0 to 1 scale methods to visualize differences in expression levels. Red star indicates seven genes with significant differences in PF/PFI. (**b**) Seven differential genes were picked for an RT-qPCR to validate transcriptome data correctness. (**c**) *PfBTB* genes’ relative expression in the stems, roots, and leaves in PF and PFI. Values are presented as the averages ± SE of three biological replicates. Each gene’s distinct letters indicate significant differences (*p* < 0.05). PF: healthy seedlings of *P. fortunei*. PFI: *P. fortunei* seedlings infected by PaWB phytoplasma.

Seven *PfBTB* genes were chosen for an RT-qPCR experiment to look at the expression levels in two separate *P. fortunei* samples in order to confirm the validity of the transcriptome sequencing analysis. As shown in Figure 5b, *PfBTB12*, *PfBTB14*, and *PfBTB44* were significantly upregulated and *PfBTB3*, *PfBTB16*, *PfBTB19*, and *PfBTB36* were significantly downregulated. Therefore, we hypothesize that these genes might have a significant impact on PaWB.

To study the roles of seven BTB genes in *P. fortunei* growth and development, we examined the expression levels of the different developmental tissues/organs. As Figure 5c illustrates, we found that the transcripts of these genes are expressed in all tissues, with *PfBTB12* showing high levels of expression in the stem and leaves of PFI, while other genes are highly expressed only in the leaves. According to the findings, the seven genes may be essential for the growth and development of *P. fortunei*.

### 2.7. Expression Patterns with Different Stress Treatments

**Figure 6:**
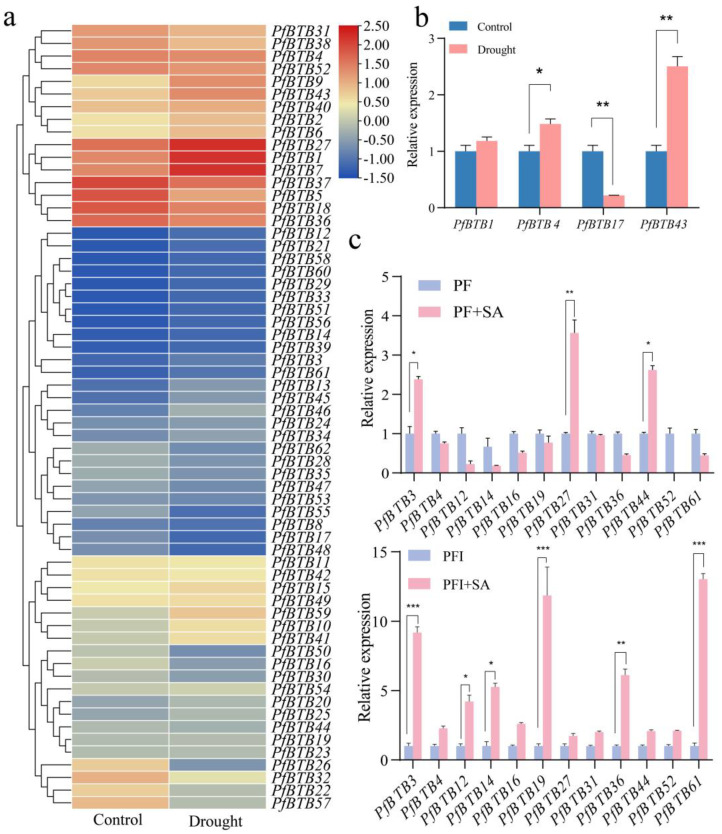
Expression analysis of *PfBTB*s under abiotic stress and hormone treatment. (**a**) Heatmap of *PfBTB* genes expressions in response to drought. The color scale represents the RPKM values normalized by log_2_(RPKM + 1). Red color represents high expression, while blue represents low expression. (**b**) RT-qPCR of *PfBTB*s genes expression in response to drought. (**c**) RT-qPCR of *PfBTB*s genes expression in response to SA treatment. The bars display the mean and standard error (*n* = 3). (* *p* < 0.05, ** *p* < 0.01 and *** *p* < 0.001).

To further confirm whether the *PfBTB* gene expression is affected by abiotic stress and hormone treatment, the expression profile of *PfBTB* under such conditions was analyzed using an RT-qPCR. We comprehensively analyzed the expression levels of *PfBTBs* during drought stress using transcriptome sequencing data. After drought treatment, *PfBTB12* and *PfBTB21* were upregulated, and *PfBTB7/14/17/19/48* was 7- to 11-fold-lower than the control, indicating that *PfBTB7/12/14/17/19/21/48* is a drought-sensitive gene (Figure 6a). We subsequently randomly selected four genes to verify the transcriptome data, and they all fit the transcriptome trend (Figure 6b). In addition, our further analysis of the evolutionary tree of the *PfBTB* gene family and the homologs of NPR1 compared the six *PfBTB3/4/27/31/52/61* genes plus the seven genes that were significantly different in PF/PFI using an RT-qPCR. The outcomes demonstrated that these gene expressions were upregulated after treatment with exogenous SA (Figure 6c). The transcriptome sequencing analysis indicated that the *PfBTB* expression was affected by drought stress, and differences in *PfBTB* expression levels suggest that they may additionally have one-of-a-kind features in response to drought stress.

### 2.8. Association Analysis of PfBTB and miRNA

**Figure 7:**
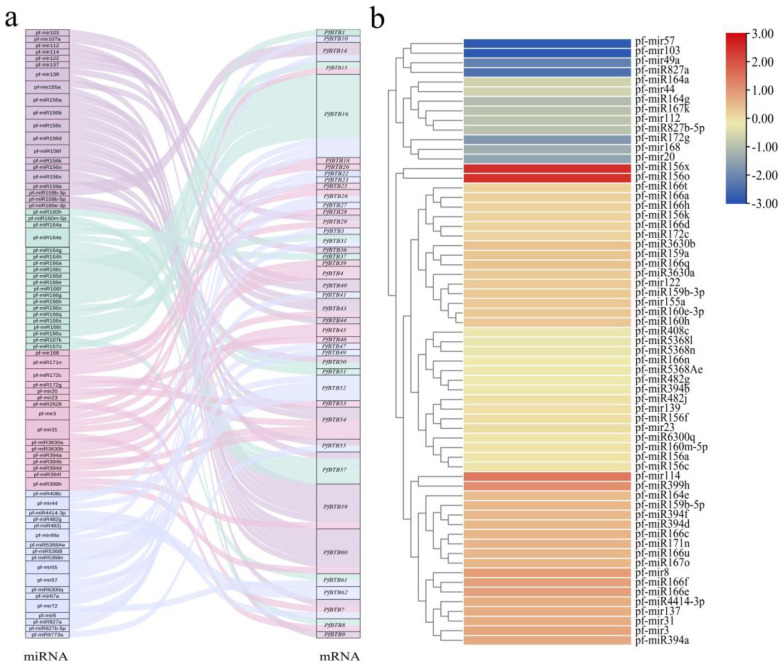
Prediction of the targeting relationship between *PfBTB*s and miRNAs and analysis of the expression of miRNAs under PaWB infection. (**a**) Sankey diagrams demonstrate the targeted binding relationship between *PfBTB*s and miRNAs. (**b**) Heatmap of miRNAs expression in response to PaWB infection. The depth of color represents the changes of the value of log2 (PFI/PF), and red represents upregulation while blue represents downregulation. PF: healthy seedlings of *P. fortunei*. PFI: *P. fortunei* seedlings infected by PaWB phytoplasma.

Using the online tool, psRNATarget, we predicted targeting relationships between the pfmiRNA sequence and the 62 members of the *PfBTB* family (Table S2) and demonstrated targeting pairs with anticipated values less than four (Figure 7a). This allowed us to confirm the regulatory role of pfmiRNA in *PfBTB* expression. The findings revealed 95 targeting relationships with expected values less than 4, including 42 members of the *PfBTB* family and 74 pfmiRNAs. The findings also revealed that the same pfmiRNA could target more than one *PfBTB* and that the same *PfBTB* could be controlled by two or more pfmiRNAs. The pf-miR166 family, which has 12 pairs of targeting relationships and 12 members, is the largest family of pfmiRNAs in this range. Ten of the twelve members of the family can simultaneously target the same *PfBTB16*. Only eight pairs of these targeting relationships are controlled by transcriptional repression, while the remaining relationships all block *PfBTB* translation into proteins via the breaking mode. The data from the PF and PFI seedlings shoots were included in the analysis of the associated pfmiRNAs (Figure 7b). The outcomes demonstrated that the expression trends of various pfmiRNAs regulating *PfBTB16* in the seedlings, pf-miR166c/pf-miR166e/pf-miR166u/pf-miR166f, were inconsistent with each other, whereas pf-miR166a/pf-miR166d/pf-miR166h/pf-miR44 had a downward trend. This suggests that the pfmiR166 family has synergistic and functional interactions and plays a significant role in controlling *PfBTB* expression.

## 3. Discussion

The number of BTB family members in plants varies greatly among different species: 69 members in grape [34], 49 members in sugar beet [35], 38 members in tomato [5], and 158 members in rice [4]. Using the recent determination of the *P. fortunei* genome, we identified 62 BTB members for the first time in *Paulownia* (Table 1). These differences in the number of BTB genes correlate with the size of the genomes of different plants, and it is likely that BTB genes have changed during the long-term evolution of plants. 

In order to classify the functional genes of the BTB family in *P. fortunei*, this research used a cluster analysis of the 62 members of *P. fortunei* together with the 80 members of *Arabidopsis thaliana*. Based on the BTB domain together with other conserved domains, 62 *PfBTB* members were classified into eight subfamilies, compared with the *Arabidopsis thaliana* BTB subfamily classification, *P. fortunei* lacks the TPR subfamily (Figure 1). We also found that the high degree of conservation of BTB family members allows genes localized in the same subfamily to have the same or similar functions. For example, the Ankyrin subfamily can respond to drought, salt stress, and ABA treatment, and *PfBTB3/4/31/32/27/52/61* in *P. fortunei* belongs to the same subfamily, so we infer that these genes may be involved in similar plant stress tolerance processes. In addition, members of the MATH subfamily have been reported to be involved in plant resistance to biotic stress processes, such as *CaBPM4* (*CaBTB51*) of the MATH subfamily of pepper, which is highly regulated under the Phytophthora capsici infection [36]. These studies suggest that within the different subfamilies of BTB, each subfamily member is involved in plant-specific biological processes.

In terms of gene structure, research has shown that gene structure (intron–exon structure) is a typical marker of gene family evolution. In this article, we found that the exon count of *PfBTB* varies greatly, ranging from 1 to 19. Among the 62 *PfBTB* genes, *PfBTB41* in the Armadillo subfamily has the most exons (19), while *PfBTB56* in the other subfamily only has one exon (Figure 2). We observed very different patterns of intron–exon structure distributions in tomato [5], grape [34], sugar beet [35], and rice [4] from those of the BTB members we studied in *Paulownia*. Thus, the differences and diversity in these structures emphasize that the evolutionary patterns between different species are complex and diverse. This gives rise to specific genomic features and regulatory mechanisms for genes.

As is well known, *cis*-acting elements are sequences that exist on gene promoters and play a crucial role in the transcriptional regulation of genes [37]. The analysis of the *cis*- acting elements of the *PfBTB* genes promoter revealed the presence of light-responsive elements, stress-responsive elements, and hormone-responsive elements (Figure 4). Among them, light-responsive elements are ubiquitous and most abundant, which is consistent with the analysis results of different species of grape [34], sugar beet [35], and tomato [5], indicating that the transcriptional regulation of PfBTB may be generally affected by light signals. Meanwhile, hormone-responsive elements, such as SA, GA, IAA, MeJA, and ABA, were also contained on the *PfBTB* gene promoter. We also found that 20 *PfBTBs* contained SA-responsive elements in the promoter region, among which members of the NPH3 subfamily were the most numerous, suggesting that NPH3 members may be involved in the transduction process of SA signals. In addition, some cold-related, defense and stress-responsive, and wound-responsive elements were also found in the promoter region of *PfBTBs*. These results suggest that *PfBTB* genes may be involved in a variety of biological processes during the growth and development of paulownias.

In addition, previous reports have shown that the BTB protein can promote plant resistance to various abiotic and biological stresses [38]. This protein affects the synthesis of related hormones in plants by participating in the Cul3 ubiquitin pathway, thereby regulating the growth, development, and stress resistance response of plants [38]. Numerous plant BTB/POZ protein family members control hormone-mediated processes and phytologically related signal network reactions, such as JA [39], SA [23,40], ABA [41,42], and GA3 [43,44], thereby regulating a series of physiological and biochemical processes including the germination of plants and metabolism, growth, and development. 

Based on the RNA-seq and RT-qPCR analyses of PF and PFI, we found that the expression of some *PfBTB* genes in *P. fortunei* was significantly influenced by PaWB (Figure 5). The expression level of *PfBTB12/14/44* increased during the formation of PaWB by *P. fortunei*, while the expression level of *PfBTB3/16/19/36* decreased. These findings indicate that the BTB family was responsive to PaWB phytoplasma infection. We know that plants synthesize a large amount of SA when infected by pathogens, and the accumulation of SA can activate the accumulation of the BTB member NPR1 in the nucleus, thereby regulating the expression of downstream defense-related genes [45,46]. Thus, we performed an RT-qPCR on the 12 selected genes, and the results showed that they were induced to be expressed in SA-treated infected seedlings. Among them, *PfBTB3/12/14/19/36* genes were significantly expressed, indicating that these genes might be crucial for the process of defense. 

Moreover, BTB genes are crucial for plant defense against abiotic stress. In *P. fortunei* under drought stress, many *PfBTB*s were stress response groups; in particular, *PfBTB12/14/19* genes were sensitive to both biotic and abiotic pressure (Figure 6a,b). MicroRNAs (miRNAs), a class of small non-coding RNAs, play significant roles in biotic and abiotic stressors by controlling their target genes [47]. According to studies on the targeting relationship between miRNAs and BTB genes, miRNA families are linked to development and stress target BTB genes [34]. By comparing the miRNA and transcriptome before and after PaWB, 74 pf-miRNAs targeting 42 *PfBTB*s were found. We found that *PfBTB14*, their target gene, was increased in diseased seedlings, while pf-miRNA159a and pf-miRNA159b-3p were downregulated in diseased seedlings [48]. Infected seedlings had increased pf-miRNA114 expression, and its *PfBTB44* target gene had increased expression as well. Therefore, we speculate that pf-miRNA114-mediated *PfBTB44* may play an important role in PaWB.

## 4. Materials and Methods

### 4.1. Plant Materials and Treatment

Tissue seedlings of PaWB-infected *P. fortunei* (PFI) and healthy *P. fortunei* (PF) were cultivated for 30 days as this study’s materials. The culture of the materials was conducted according to the method by FAN et al. [49]. For hormone treatment, the PFI seedlings were treated by adding 0.2 mM SA to the medium without SA as a control. For drought treatment seedlings (PF), the soil water content was 25%, the soil water content was 75% as control, and the second round of leaves were collected for 30 days during the drought stress. The samples were collected and stored in −80 °C.

### 4.2. Identification of BTB Gene Family Members in P. fortunei’s Genome

To discover the workable members of the BTB gene, we first downloaded the *P. fortunei* genome from NCBI (https://www.ncbi.nlm.nih.gov/ (accessed on 27 June 2022)). Then, using the HMM search software (v. 3.1), we searched for the putative BTB genes in the *P. fortunei* protein database using the seed file for the BTB domain (PF00651). To check the accuracy of the BTB conserved domain, all candidate proteins were then uploaded to the SMART website (http://smart.embl-heidelberg.de/ (accessed on 29 June 2022)). Additionally, the *PfBTB* proteins’ pI and MW were determined using ExPASy ProtParam (accessed on 13 July 2022), an online analysis tool. Furthermore, we investigated the subcellular locations of PfBTBs using the WoLF PSORT website (https://www.genscript.com/wolf-psort.html (accessed on 23 July 2022)).

### 4.3. PfBTB Proteins Underwent Phylogenetic Analysis

The BTB protein sequences of *Arabidopsis thaliana* were downloaded from TAIR (https://www.Arabidopsis.org/browse/genefamily/pub.jsp (accessed on 27 July 2022)). All BTB protein sequences of *P. fortunei* and *Arabidopsis* were compared using ClustalW. MEGA-X 10.2 (Method, NJ; Bootstrap, 1000) was then used to build the phylogenetic tree. Finally, the evolutionary tree of the BTB proteins was modified using the iTOL online website, (https://itol.embl.de/ (accessed on 30 August 2022)).

### 4.4. Analysis of Gene Structures, Motifs, and Cis-Acting Elements

Gene structure data was taken from the *P. fortunei* whole genome database in order to identify and represent the introns, exons, and UTR structural organization of the *PfBTB* gene family. The unique conserved motifs of *PfBTB* genes were discovered using the MEME suite (http://meme-suite.org/tools/meme (accessed on 13 September 2022)). For the analysis, 20 motifs were used in total, with a maximum of 200 amino acids for motif width. TBtools (v. 2.019) was used to visualize the results of the gene structure and conserved motif analyses [50]. 

The 2000 bp upstream location of *PfBTB*s was once described as a putative promoter sequence. We obtained such sequences of *PfBTB*s using TBtools (v. 2.019). *Cis*-acting factors of the putative promoter location of these genes were estimated using PlantCare (https://bioinformatics.psb.ugent.be/webtools/plantcare/html/ (accessed on 27 September 2022)) [51]. According to the characteristic annotations of the *cis*-acting elements, the intriguing portions have been saved for later research, and the *cis*-acting element using equal functional annotations were built-in into the equal group.

### 4.5. Chromosomal Localization and Synteny Analysis

*PfBTB* homologous gene pairs were discovered using an all-vs-all blast method and blast software. The synteny areas were then determined by MCScanX program utilizing the all-vs-all blast results. To display the distribution of synteny gene pairs, we plotted circus images. TBtools (v. 2.019) carried out the analysis of the chromosomal locations [50].

### 4.6. Prediction of the miRNA and PfBTB Targeting Relationship

Using the psRNATarget online software (https://www.zhaolab.org/psRNATarget/ (accessed on 15 March 2023)), the regulatory link between *PfBTB* and pfmiRNA was predicted with a mismatch value of one, an expectation value less than or equal to four, and other analytic parameters set to the system default [52]. Tbtools (v. 2.019) was used to evaluate and create graphs from the transcriptome data of all predicted pfmiRNAs in sick seedlings.

### 4.7. Investigation of PfBTB Expression Patterns

The RNA-seq data under different treatments of *P. fortunei* were obtained from our previous study [27]. We downloaded these data from NCBI (accession number: SRR11787938, SRR11787927, SRR11787916, SRR11787905, SRR11787894, SRR11787883, PRJNA221355) [53]. Then, heat maps were plotted using TBtools (v. 2.019).

The PF and PFI samples’ total RNA was extracted using a total RNA extraction kit. (Tiangen Biotech Co., Beijing, China). Then, the RNA was reverse-transcribed into cDNA using the PrimeScriptTM RT reagent kit (GenStar, Beijing, China). A quantitative real-time PCR (RT-qPCR) analysis was undertaken using a 2×RealStar Fast SYBR qPCR Mix (Low ROX) (GenStar, Beijing, China). With the aid of the Primer 5.0 software, we created fifteen pairs of precise primers, which were then validated using NCBI (Table S1). The relative expression of genes was determined using the 2^−∆∆CT^ technique with *actin* as an internal reference. 

### 4.8. Statistical Analysis

All results were collected from three parallel experiments. By using the analysis of variance (ANOVA) and Duncan’s multiple range test with significant differences (*p* < 0.05), data were compared with the control group or between treatments. For the Student’s *t* test, * *p* < 0.05; ** *p* < 0.01, and *** *p* < 0.001. Graphs were plotted using GraphPad Prism 8.0.

## 5. Conclusions

We found 62 *PfBTB* members of the *P. fortunei* genome in this investigation. Based on the phylogenetic tree analysis, nine groups of this species’ BTB gene family were discovered. Analyses of conserved motifs and gene structures offered compelling support for the classification outcomes obtained from the phylogenetic trees. The *cis*-acting element analysis revealed that the BTB family genes contain various sensitive elements that allow *P. fortunei* to respond to biotic and abiotic factors. The transcriptional sequencing data analysis revealed different expression levels of the *PfBTB* gene. Candidate genes implicated in biotic and abiotic stressors were subsequently identified using an RT-qPCR. Our analysis provides a solid foundation for future studies on the molecular mechanisms of *PfBTB* genes in response to biotic and abiotic stresses.

## Figures and Tables

**Table 1:** Information on the *P. fortunei* BTB gene family.

Gene Name	Gene ID	Chromosome	Protein Length (aa)	Molecular Weight (Da)	Theoretical pI	Putative Subcellular Localization
*PfBTB1*	Pfo02g001220	Chr2	564	64,356.53	5.17	nucl: 11, chlo: 1, plas: 1, golg: 1
*PfBTB2*	Pfo02g015880	Chr2	625	69,264.02	6.7	nucl: 7, cyto: 6, mito: 1
*PfBTB3*	Pfo03g001400	Chr3	486	52,888.35	6.14	chlo: 5, cyto: 5, nucl: 3, plas: 1
*PfBTB4*	Pfo03g008270	Chr3	587	65,515.92	5.78	cyto: 4, nucl: 3, E.R.: 3, vacu: 2, chlo: 1,
*PfBTB5*	Pfo03g010830	Chr3	496	55,425.03	4.64	nucl: 12, cyto: 1, pero: 1
*PfBTB6*	Pfo04g002970	Chr4	626	69,187.25	7.53	nucl: 4, cyto: 4, chlo: 3, mito: 2, cysk: 1
*PfBTB7*	Pfo04g013910	Chr4	572	64,898.01	5.07	nucl: 10, chlo: 2, cyto: 1, plas: 1
*PfBTB8*	Pfo05g002190	Chr5	623	69,677.81	8.84	chlo: 6, cyto: 4, nucl: 3, mito: 1
*PfBTB9*	Pfo05g002850	Chr5	410	45,607.9	6.17	chlo: 6, mito: 3, nucl: 2, cyto: 2, plas: 1
*PfBTB10*	Pfo05g003240	Chr5	666	73,530.9	5.62	cyto: 11, nucl: 2, cysk: 1
*PfBTB11*	Pfo05g007860	Chr5	403	44,661.49	5.68	cyto: 9, nucl: 3, extr: 1, vacu: 1
*PfBTB12*	Pfo05g013850	Chr5	632	71,008.93	7.25	nucl: 4, cyto: 4, chlo: 3, mito: 1, plas: 1,
*PfBTB13*	Pfo05g016000	Chr5	607	68,367.01	6.25	vacu: 7, chlo: 2, nucl: 2, mito: 1, plas: 1,
*PfBTB14*	Pfo06g002150	Chr6	639	72,922.16	7.54	nucl: 9, chlo: 3, mito: 1, pero: 1
*PfBTB15*	Pfo06g010190	Chr6	379	43,425.7	9.09	cyto: 6, nucl: 5, chlo: 1, plas: 1
*PfBTB16*	Pfo06g010240	Chr6	605	67,284.45	5.58	cyto: 8, nucl: 3, chlo: 2, cysk: 1
*PfBTB17*	Pfo06g010330	Chr6	619	68,873.48	6	cyto: 5.5, cyto_nucl: 5, nucl: 3.5, chlo: 2
*PfBTB18*	Pfo07g012470	Chr7	544	61,639.22	5.23	nucl: 9, cyto: 2, plas: 1, cysk: 1, golg: 1
*PfBTB19*	Pfo08g000530	Chr8	393	44,326.69	5.94	nucl: 6, cyto: 6, chlo: 1, vacu: 1
*PfBTB20*	Pfo08g000540	Chr8	365	41,226.8	7.54	chlo: 8, mito: 5.5, cyto_mito: 3.5
*PfBTB21*	Pfo08g000630	Chr8	227	24,879.48	6.35	mito: 6.5, cyto_mito: 4, nucl: 3, chlo: 2
*PfBTB22*	Pfo08g000660	Chr8	396	44,387.72	5.65	chlo: 8.5, chlo_mito: 5.5, nucl: 3, mito: 1.5
*PfBTB23*	Pfo08g000690	Chr8	411	46,884.72	7.21	nucl: 7, mito: 6, chlo: 1
*PfBTB24*	Pfo08g000720	Chr8	394	44,389.13	6.48	cyto: 9, nucl: 3, mito: 2
*PfBTB25*	Pfo08g000730	Chr8	382	42,939.41	7.21	chlo: 12, mito: 1.5, cyto_mito: 1.5
*PfBTB26*	Pfo08g001180	Chr8	358	41,041.9	9.52	nucl: 8, mito: 3, plas: 1.5, golg_plas: 1.5
*PfBTB27*	Pfo09g000940	Chr9	586	65,718.53	6.57	cyto: 4, E.R.: 4, golg: 2, chlo: 1, nucl: 1
*PfBTB28*	Pfo09g001930	Chr9	602	66,580.42	5.78	nucl: 5, cyto: 4, chlo: 3, plas: 1, cysk: 1
*PfBTB29*	Pfo09g009710	Chr9	1016	115,074.68	6.61	plas: 6, E.R.: 5, nucl: 1, mito: 1, pero: 1
*PfBTB30*	Pfo09g010190	Chr9	328	37,502.9	5.63	cyto: 8, nucl: 4, mito: 1, plas: 1
*PfBTB31*	Pfo09g019460	Chr9	603	67,411.24	6.07	chlo: 8, cyto: 3, nucl: 2, extr: 1
*PfBTB32*	Pfo09g020220	Chr9	596	67,308.42	4.92	nucl: 10.5, cyto_nucl: 6.5, cyto: 1.5, chlo: 1
*PfBTB33*	Pfo10g006800	Chr10	270	30,234.37	5.59	nucl: 7, chlo: 4, mito: 2, cyto: 1
*PfBTB34*	Pfo10g012330	Chr10	616	69,548.02	5.6	chlo: 8, nucl: 2, cyto: 2, plas: 1, extr: 1
*PfBTB35*	Pfo11g007120	Chr11	401	45,685.27	9.1	nucl: 6, cysk: 3, chlo: 2, cyto: 2, plas: 1
*PfBTB36*	Pfo11g008220	Chr11	804	92,144.78	5.44	E.R.: 4, nucl: 3, cyto: 3, plas: 3, chlo: 1
*PfBTB37*	Pfo11g014130	Chr11	256	28,694.63	4.9	chlo: 6, cyto: 4, mito: 2, nucl: 1, plas: 1
*PfBTB38*	Pfo11g014430	Chr11	327	37,633.11	5.62	cyto: 9, chlo: 1, nucl: 1, mito: 1, cysk: 1
*PfBTB39*	Pfo12g001080	Chr12	633	70,819.11	9.13	cyto: 6, chlo: 5, nucl: 2, mito: 1
*PfBTB40*	Pfo12g001360	Chr12	410	45,439.7	6.07	chlo: 3, nucl: 3, cyto: 3, mito: 3, plas: 1
*PfBTB41*	Pfo12g001600	Chr12	709	78,578.93	6.22	cyto: 7, nucl: 4, mito: 1, vacu: 1, cysk: 1
*PfBTB42*	Pfo12g004960	Chr12	404	44,534.33	5.88	cyto: 7, nucl: 4, chlo: 1, mito: 1, cysk: 1
*PfBTB43*	Pfo12g006350	Chr12	641	72,314.29	8.01	cyto: 3, chlo: 2, nucl: 2, mito: 2, vacu: 2
*PfBTB44*	Pfo12g010520	Chr12	563	63,201.63	7.9	plas: 9, vacu: 2, nucl: 1, extr: 1, E.R.: 1
*PfBTB45*	Pfo12g011820	Chr12	607	68,499.04	6.03	nucl: 6, cysk: 3, chlo: 2, cyto: 1, mito: 1
*PfBTB46*	Pfo13g007700	Chr13	555	62,398.01	8.3	chlo: 7, nucl: 5, cyto: 1, golg_plas: 1
*PfBTB47*	Pfo13g007770	Chr13	545	60,899.48	5.5	cyto: 7, nucl: 3, chlo: 2, mito: 1, vacu: 1
*PfBTB48*	Pfo13g007850	Chr13	606	68,444.12	7.91	nucl: 8, cyto: 4, chlo: 1, cysk: 1
*PfBTB49*	Pfo14g010540	Chr14	399	45,657.17	9	nucl: 13, cyto: 1
*PfBTB50*	Pfo15g002860	Chr15	345	39,347.06	5.25	cyto: 6, nucl_plas: 3, nucl: 2.5, plas: 2.5
*PfBTB51*	Pfo15g003440	Chr15	292	33,412.69	5.08	nucl: 6, mito: 4, chlo: 3, cysk: 1
*PfBTB52*	Pfo16g000510	Chr16	588	65,792.84	5.99	cyto: 6, nucl: 4, chlo: 1, mito: 1, plas: 1
*PfBTB53*	Pfo16g008780	Chr16	328	37,300.61	5.39	cyto: 7, nucl: 2, mito: 2, plas: 2, pero: 1
*PfBTB54*	Pfo16g013570	Chr16	633	70,396.02	5.94	nucl: 6, chlo: 4, cyto: 2, plas: 1, cysk: 1
*PfBTB55*	Pfo18g003810	Chr18	617	68,606.8	8.82	cyto: 6, nucl: 4, vacu: 2, chlo: 1, E.R.: 1
*PfBTB56*	Pfo18g011610	Chr18	428	48,041.23	4.82	chlo: 4, nucl: 3.5, cysk_nucl: 2.5, plas: 2
*PfBTB57*	Pfo19g001900	Chr19	857	94,729.13	5.59	nucl: 12, cyto: 1, vacu: 1
*PfBTB58*	Pfoxxg008290	Contig01580	616	68,975.67	5.67	cyto: 8, nucl: 5, vacu: 1
*PfBTB59*	Pfoxxg012560	Contig01619	383	42,468.21	5.71	nucl: 8, chlo: 4, cyto: 1, cysk: 1
*PfBTB60*	Pfoxxg012630	Contig01622	312	34,410.13	6.19	nucl: 8.5, cyto_nucl: 6, chlo: 3, cyto: 2.5
*PfBTB61*	Pfoxxg018320	tig00016041	452	49,057.37	6.05	cyto: 6, chlo: 5, nucl: 1, plas: 1
*PfBTB62*	Pfoxxg021290	tig00016941	549	61,153.44	6.11	chlo: 10.5, chlo_mito: 6, nucl: 3

## Data Availability

Data are contained within the article and Supplementary Materials.

## Supplementary Materials

The following are available online at https://www.mdpi.com/article/10.3390/plants12244144/s1, Table S1: Primer sequence for RT-qPCR. Table S2: Prediction of targeting relationship between pfmiRNA and *PfBTB*.
